# A Mathematical Model of Neutral Lipid Content in terms of Initial Nitrogen Concentration and Validation in* Coelastrum* sp. HA-1 and Application in* Chlorella sorokiniana*

**DOI:** 10.1155/2017/9253020

**Published:** 2017-01-18

**Authors:** Zhenhua Yang, Yue Zhao, Zhiyong Liu, Chenfeng Liu, Zhipeng Hu, Yuyong Hou

**Affiliations:** Tianjin Key Laboratory for Industrial Biological Systems and Bioprocessing Engineering, Tianjin Institute of Industrial Biotechnology, Chinese Academy of Sciences, Tianjin, China

## Abstract

Microalgae are considered to be a potential major biomass feedstock for biofuel due to their high lipid content. However, no correlation equations as a function of initial nitrogen concentration for lipid accumulation have been developed for simplicity to predict lipid production and optimize the lipid production process. In this study, a lipid accumulation model was developed with simple parameters based on the assumption protein synthesis shift to lipid synthesis by a linear function of nitrogen quota. The model predictions fitted well for the growth, lipid content, and nitrogen consumption of* Coelastrum* sp. HA-1 under various initial nitrogen concentrations. Then the model was applied successfully in* Chlorella sorokiniana* to predict the lipid content with different light intensities. The quantitative relationship between initial nitrogen concentrations and the final lipid content with sensitivity analysis of the model were also discussed. Based on the model results, the conversion efficiency from protein synthesis to lipid synthesis is higher and higher in microalgae metabolism process as nitrogen decreases; however, the carbohydrate composition content remains basically unchanged neither in HA-1 nor in* C. sorokiniana*.

## 1. Introduction

Microalgae have been considered as a potential biomass feedstock for renewable energy technologies due to its high cellular concentration of lipids, resources sustainability, and greater potential environmental benefits than conventional biofuel [1–3]. However, realizing industrial production of algal-derived biofuel faces many obstacles; in particular, the cost and technical constraints are the two currently major limits for widespread adoption of algae oil [4–7]. Therefore, algae-derived oil development may require progress in various scientific and engineering disciplines, such as optimizing cultivation parameters to increase lipid productivities [4, 8, 9]. Considering the assumption that the algae biomass (dry weight) was the sum of protein, lipids, and carbohydrates [5], the lipid productivity could be affected by protein and carbohydrate synthesis. Although the excess carbon fixed by photosynthesis is changed to triacylglycerides synthesis for lipid accumulation under nitrogen starvation, the growth rate of microalgae is reduced since fundamental proteins cannot be synthesized [5, 10–12]. On the other hand, mechanism on neutral lipid synthesis about how TAGs are biosynthesized is still in its early stage. Li et al. and Hong et al. [13, 14] observed the competitive relationship between TAGs and carbohydrates biosynthesis after nitrogen depletion. Interestingly, some strains show significant higher quantities of both carbohydrates and lipid during nitrogen-deprived condition [15, 16]. For this reason a suitable way to increase both lipid content and biomass is an urgent and pressing need for producing microalgal biofuels.

The growth model of algae has been well developed with various algae species under different growth environment [17–20]. However, the easily adaptable model of algal lipid content is rarely published with respect to various nitrogen concentrations. A new widely simplified applicable mathematical model should be developed for various nitrogen treatments to fully understand the microalgae lipid productivity.

In this study, the lipid content was represented as a linear function of nitrogen quota which was calculated based on the mass balance in the growth medium to study lipid accumulation. It was validated in* Coelastrum* sp. HA-1 under various initial nitrogen concentrations and applied in* Chlorella sorokiniana* at different light conditions. The growth was modeled by a nonautonomous differential equation incorporating the logistic equation and adjustment function [14, 15], whereas Michaelis-Menten function was used to model the nitrogen uptake [18, 19].

## 2. Methods

### 2.1. Algae Growth Kinetic Modeling

In order to obtain the relationship between culture conditions and the specific growth rate, a simple but useful model (1) was expressed in this work by a nonautonomous differential equation [21–23].

(1) $$\begin{array}{c} \frac{1}{X\left(t\right)}\cdot \frac{dX\left(t\right)}{dt}=\mu =\mu_{max}\cdot \alpha \left(\;t\;\right)\cdot \left(\;1-\frac{X\left(t\right)}{X_{max}}\;\right), \end{array}$$

where *μ* is the specific growth rate of cell population (day^−1^), *X*(*t*) is the microalgal concentration at time *t* (g dw·L^−1^), *μ*_max_ is the maximum specific growth rate of cells (day^−1^), *α*(*t*) is the adjustment function, and *X*_max_ is the maximum microalgal concentration during cultivation (g dw·L^−1^).

Baranyi and Roberts [21, 22] reported *α*(*t*) based on an assumption that the growth of cells in the lag phase was inhibited by intracellular substance. The adaptation of microalgal cells can be described by *α*(*t*) when cells entered in a new condition of the bioreactor. Adjustment function  is expressed as

(2) αt=exp⁡−h0exp⁡−μmax·t+exp⁡−h0−exp⁡−μmax·t−h0,

where *h*_0_ is called the dimensionless parameter of Baranyi-Roberts model. Adjustment function has the following characteristics: it is a monotonic function; lim_*t*→*∞*_⁡*α*(*t*) = 1; when *h*_0_ < 0, *α*(*t*) > 1; when *h*_0_ ≥ 0, 0 ≤ *α*(*t*) < 1.

### 2.2. Nitrogen Assimilation and Nitrogen Quota Modeling

In this work, we assumed that nitrogen uptake is a function of external nitrogen concentration; thus, Michaelis-Menten function [18, 19] was used to simulate nitrogen uptake during cultivation.

The differential equation for nitrogen uptake is expressed as

(3) $$\begin{array}{c} \frac{dN\left(t\right)}{dt}=-v_{m}\cdot \frac{N\left(t\right)}{N\left(t\right)+K_{N}}\cdot X\left(\;t\;\right), \end{array}$$

where *N*(*t*) is the external nitrogen concentration at time *t* (g N·L^−1^), *v*_*m*_ is the maximum nitrogen uptake rate (g N g^−1^ dw·day^−1^), and *K*_*N*_ is the half-saturation coefficient of nitrogen uptake (g N·L^−1^).

Integration of (3) yields the remaining nitrogen in the growth media at time *t*. The nitrogen quota that is defined as the rate between the mass of internal nitrogen and total dry weight of biomass can be obtained by mass balance [24]. It is expressed as

(4) $$\begin{array}{c} Q\left(\;t\;\right)=\frac{N_{0}-N\left(t\right)+X_{0}\cdot Q_{0}}{X\left(t\right)}, \end{array}$$

where *Q*(*t*) is the nitrogen quota at time *t* (g N g^−1^ dw), *N*_0_ is the initial nitrogen concentration in growth media (g N·L^−1^), *Q*_0_ is the initial nitrogen quota (g N g^−1^ dw), and *X*_0_ is the initial microalgal concentration (g dw·L^−1^).

### 2.3. Lipid Accumulation Modeling

The conversion efficiency from protein synthesis to lipid or carbohydrate synthesis is higher and higher in microalgae metabolism process when nitrogen is depleted causing a change in the biomass composition [20, 25]. Once nitrogen is depleted, lipid content increased from 9 percent to 62 percent of dry weight; however, protein content decreased from 53 percent to 23 percent of dry weight, and carbohydrate content increased a little in the biomass [20]. The lipid accumulation model thus was expressed as (5) by assuming that the biomass is made up of protein, lipid, and carbohydrate, and protein synthesis shifts to lipid synthesis with nitrogen depletion.

(5) $$\begin{array}{c} L\left(\;t\;\right)=1-k\cdot Q\left(\;t\;\right)-c, \end{array}$$

where *L*(*t*) is the lipid content (g Lipid g^−1^ dw), *k* is the nitrogen-to-protein conversion factor, and *c* is the carbohydrate content of biomass (g carbohydrate g^−1^ dw).

After rearranging (4)-(5) and replacing *X*_0_. *Q*_0_ with Φ, the lipid content becomes

(6) $$\begin{array}{c} L\left(\;t\;\right)=1-k\cdot \frac{N_{0}-N\left(t\right)+\Phi }{X\left(t\right)}-c. \end{array}$$

We could easily obtain the lipid content according to (6) by analyzing nitrogen concentrations and microalgal biomass.

### 2.4. Experimental Approach

The seeds of* Coelastrum* sp. and* C. sorokiniana* were cultured to logarithmic phase for further use in 250 mL Erlenmeyer flasks with 150 mL F medium (nutrient elements are twofold compared to those in f/2 medium) and Kuhl medium under continuous illumination (100 *μ*mol·m^−2^·s^−1^), respectively [26, 27]. The temperature was maintained at 25°C; pH was adjusted to 7.5 initially.

In order to evaluate the effect of nitrogen on the dry weight and lipid yield of the* Coelastrum* sp. within 24 days, the F medium with 150 *μ*mol·m^−2^·s^−1^ was modified by adjusting NaNO_3_ concentrations to 0.075, 0.15, 0.3, 0.6, and 0.9 g·L^−1^. For model application on fresh water microalgae* C. sorokiniana*, the cells were cultivated in modified Kuhl medium with 1 g·L^−1^ NaNO_3_ under different light intensities (50, 100, and 200 *μ*mol·m^−2^·s^−1^). In the experiments, 50 mL logarithmic phase seed cells were centrifuged and resuspended with the corresponding medium to wipe out the effect of residual nitrogen source and then transferred to glass columns (4.1 cm in diameter, 37 cm in height) with 2% (v/v) CO_2_ at 25°C. The cell concentration (OD_680_) of each column reactor was approximately 0.2. For dry weight measurement, microalgal culture sample (10 mL) was filtered through pretreated glass-fiber filter paper (0.8 *μ*m pore size); then 0.5 mole solution of ammonium formate was used to wash the filter paper in order to remove salt. Finally, the filter paper was dried 24 h at 105°C [28, 29]. The dry weight was calculated by the difference between the cell-containing dried filter paper and the weight before filtration. The lipid content and nitrogen concentration were measured according to Yuan et al.'s method [29]. All experiments were performed with triplicates.

### 2.5. Parameter Estimation

In order to determine the parameter values of (6), the MATLABlsqcurvefit routine was firstly used to estimate the parameter values of (1) and (3) which were integrated by Runge-Kutta integration method. Then, (6) was used for fitting the experimental data of lipid content under different initial nitrogen concentrations. For HA-1, the parameter values and simulation *R*^2^ were shown at different initial nitrogen concentrations in Table 1. The parameter values of the model for predicting growth, lipid content, and nitrogen uptake of* Chlorella sorokiniana* were in Table 2.

## 3. Results and Discussion

### 3.1. Effect of Initial Nitrogen Concentration on the Final Lipid Content in* Coelastrum* sp. HA-1

**Figure 1:**
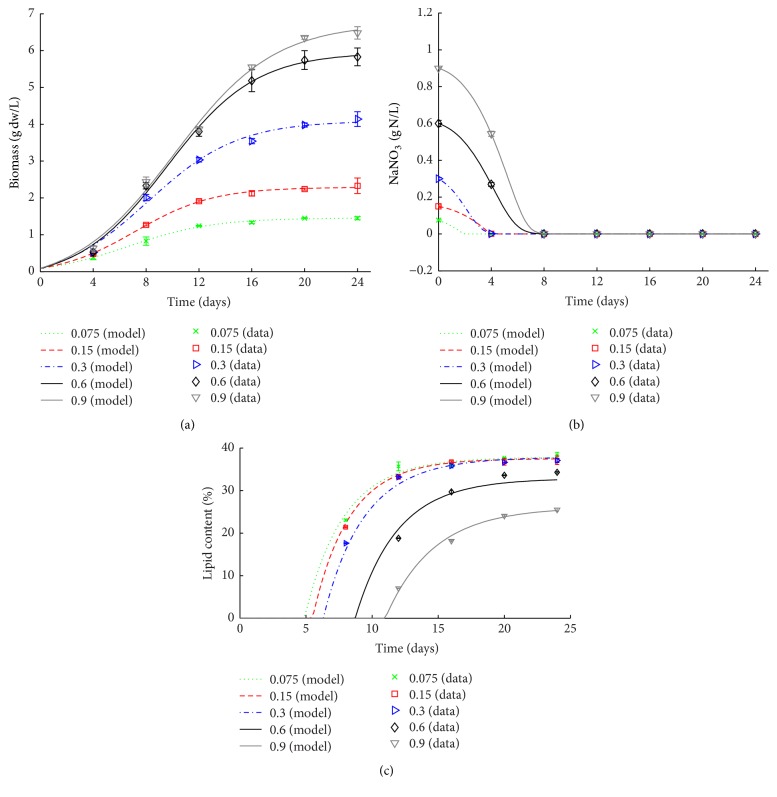
Simulation results versus experimental data for dry weight (a), nitrogen consumption (b), and lipid content (c) of* Coelastrum* sp. HA-1 at different initial NaNO_3_ concentrations.

The final lipid content of HA-1 under low initial nitrogen concentration was higher than those with high nitrogen concentration (Figure 1(c)). It might be explained from the point of energy balance that cell growth will not continue under low nitrogen concentration. However, carbon fixation is still in progress at rates exceeding the needs of the cell. In order to maintain a safe turnover of the ATP and reductant pools sustained by light reaction, neutral lipid synthesis is upregulated because fatty acid production needs more ATP and reductant than other substance production in cell; neutral lipids store significantly more energy and its synthesis requires twice energy than carbohydrate or protein [30].

In order to quantify the relationship between initial nitrogen concentration and the final lipid content in HA-1, a correlation equation of *X*_max_ was firstly given by polynomial fitting with initial nitrogen concentrations. The empirical correlation equation of *X*_max_ was

(7) $$\begin{array}{c} X_{max}=-7.891\cdot N_{0}^{2}+14.070\cdot N_{0}+0.439. \end{array}$$

**Figure 2:**
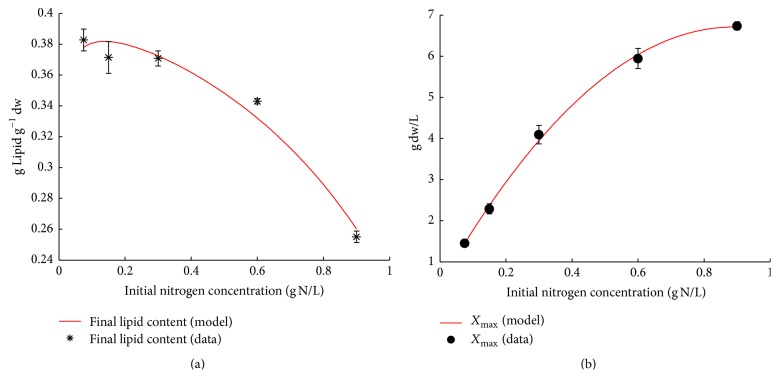
Simulation results versus parameter values for the final lipid content of* Coelastrum* sp. HA-1 (a) and the maximum algal concentration (b) under different initial NaNO_3_ concentrations.

Based on (6), the final lipid content could be calculated by initial nitrogen concentration when algal concentration reached the maximum value. After rearranging (6) and (7), the final lipid content could be expressed as

(8) $$\begin{array}{c} L_{f}=0.56-\frac{2.120\cdot \left(N_{0}+0.0495\right)}{-7.891\cdot N_{0}^{2}+14.070\cdot N_{0}+0.439}, \end{array}$$

where *L*_*f*_ is the final lipid content (g Lipid g^−1^ dw). The simulation result of (8) is meaning for final lipid content data (Figure 2(a)).

The simulation of (7) agreed with the initial nitrogen concentration data to a great degree (Figure 2(b)), and the simulation *R*^2^ was 0.9982. Equation (7) showed that the relationship between *X*_max_ and initial concentration of nitrogen was not linear, especially when the initial concentration of nitrogen was higher in growth medium (Figure 2(b)). Compared to high nitrogen conditions, the maximum microalgal concentration increased significantly with the nitrogen concentration increasing under low nitrogen conditions. The biomass with 0.3 g·L^−1^ nitrogen was about 1.86-fold compared to those in 0.075 g·L^−1^ nitrogen medium. While the nitrogen increased from 0.6 to 0.9 g·L^−1^, the maximum microalgal concentration was only increased about 11.1%. Although the growth decreased with nitrogen concentration increasing, the final microalgal concentration increased to 6.48 g·L^−1^. The results strongly imply that growth is limited by another nutrient or light intensity when BG-11 is rich in nitrogen. A similar result was also reported by Packer et al. [24].

Equation (8) showed that the final lipid content could be described as a function of initial nitrogen concentration. Decreasing the initial nitrogen concentration is a good method to obtain high lipid production especially for outdoor cultivation. It is important to note that the range of initial concentration of nitrogen for artificial regulation needs to be obtained based on experimental data under a certain cultivation condition. Based on (7) and (8), a two-stage cultivation process may be an effective way to reach great lipid productivity, similar to the previous reports [31–34]. The first stage increases biomass by controlling high initial concentration and then improves the lipid content by controlling low initial nitrogen concentration in lipid accumulation stage. This model also can be used for other microalgal species by adjusting parameter values.

### 3.2. Models Validation in HA-1

The simulation results of (1)–(6) for dry weight (Figure 1(a)), NaNO_3_ concentration (Figure 1(b)), and lipid content (Figure 1(c)) were in agreement with the experimental data. With the increase of initial nitrogen concentration, the maximum specific growth rate was basically stable. In order to explain the relationship between *h*_0_ and initial NaNO_3_ concentration, we fit *h*_0_ and initial NaNO_3_ concentration (*N*_0_) to build a linear model: *h*_0_ = −1.09*N*_0_ − 0.6711. Meanwhile, *α*(*t*) is a monotonic function, lim_*t*→*∞*_⁡*α*(*t*) = 1, when *h*_0_ < 0, *α*(*t*) > 1. Hence, *h*_0_ can indicate the grow rate of microalgae in difference initial NaNO_3_ concentrations. The value of *h*_0_ was negative at different nitrogen concentrations and trended to decrease with an increase of the initial concentration of nitrogen. Figure 1(a) showed the mathematical model fitting of growth with the average of repeated experimental values. For low nitrogen levels (0.075 and 0.15 g·L^−1^ NaNO_3_), the short logarithmic phase resulted in low cell density. However, in 0.6 and 0.9 g·L^−1^ NaNO_3_ groups, the logarithmic phase of the cells exceeds 20 days and the maximum biomass was 6.48 g·L^−1^ at 0.9 g·L^−1^ initial NaNO_3_ concentration. Figure 1(b) showed that NaNO_3_ with initial concentrations of 0.075, 0.15, and 0.3 were absolutely depleted at the 4th day and with 0.6 and 0.9 g·L^−1^ were depleted at the 8th day. Meanwhile, lipid content was increasing sharply when NaNO_3_ was depleted and subsequently became stable (Figure 1(c)). The results and (7) imply that biomass and lipid accumulation can be controlled by nitrogen supply in a certain range in terms of other conditions unchanged. However, the varying uncontrollable culture conditions for large scale open outdoor restricted the application. Thus, we further discussed application of light, one of the restricting factors, in our model.

The maximum lipid content was 38% which was reached in 0.075 g·L^−1^ NaNO_3_ after 24 days. As initial concentration of NaNO_3_ is increasing, the final lipid content decreased from 38% to 25% (Figure 1(c)). These results indicate that nitrogen quota is the key factor for lipid accumulation, especially on the nitrogen limitation condition. The similar results were also reported in previous literatures [24, 35–37].

As illustrated in Table 1, a simple sensitivity analysis for parameters of the model was performed based on two repeats, and all of the parameter values had a range for the model. The sensitivity results show that the model is stable for HA-1 in two repeats.

### 3.3. Model Application for* C. sorokiniana* under Photoautotrophic Condition

In order to extend application of the model in other microalgal species under various cultivation conditions, the model was used to predict the growth, NaNO_3_ consumption, and lipid accumulation of the* C. sorokiniana* under phototrophic cultivation condition.

Based on these experimental data, the model was used to predict the growth, lipid content, and NaNO_3_ consumption (Table 2). The values of the nitrogen-to-protein conversion factor, the carbohydrate content of biomass, and Φ were estimated according to the final lipid content of* C. sorokiniana*. The parameters *v*_*m*_ and *K*_*N*_ of* Coelastrum* sp. HA-1 at 0.9 g·L^−1^ initial NaNO_3_ concentration were used directly; then the final lipid content and the biomass data of* C. sorokiniana* at different light intensity were as target to estimate *μ*_max_, *h*_0_, *k*, *c*, and Φ using (1), (3), and (6). In addition, the sensitivity analysis was analyzed in Section 3.5.

**Figure 3:**
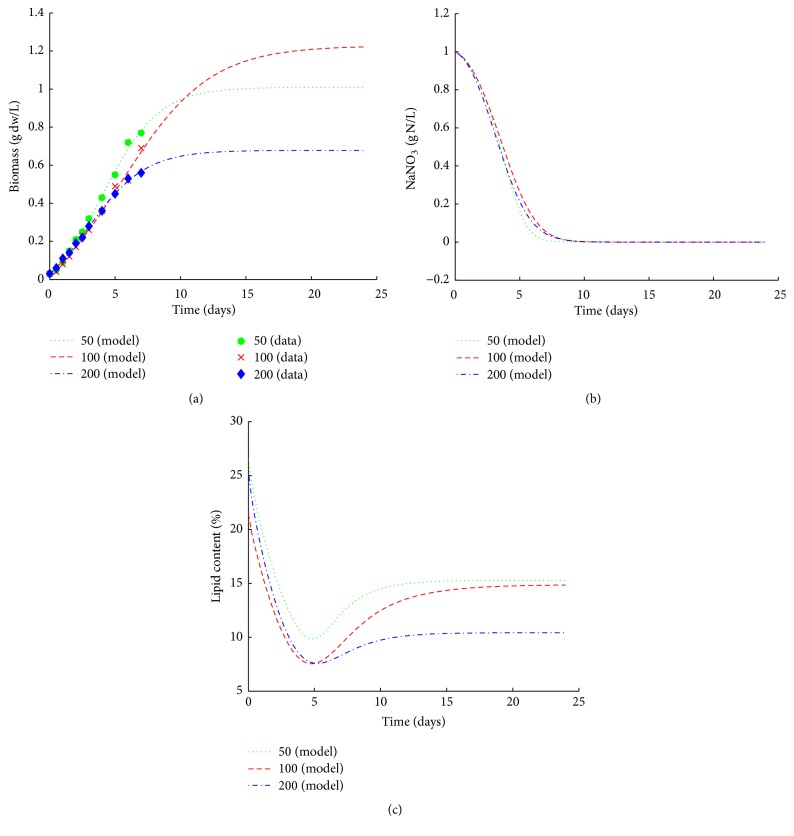
Simulation results of* C. sorokiniana* versus experimental data for dry weight (a) and predictions of nitrogen consumption (b) and lipid content (c) at different light intensities supplemented.

The growth of* C. sorokiniana* was slow (Figure 3(a)) under phototrophic cultivation condition. After 7 days, the maximum dry weight (0.77 g·L^−1^) for* C. sorokiniana* was reached under 50 *μ*mol·m^−2^·s^−1^ of light intensity. The data of NaNO_3_ consumption (Figure 3(b)) of* C. sorokiniana* were similar to HA-1. According to the predictions, NaNO_3_ was completely depleted around days 12, 12, and 15 at 50, 100, and 200 *μ*mol·m^−2^·s^−1^, respectively.

The predictions of lipid content of* C. sorokiniana* were shown in Figure 3(c) under light intensity of 50, 100, and 200 *μ*mol·m^−2^·s^−1^. During logarithmic phase, the lipid content of* C. sorokiniana* decreased for three types of light conditions. The lipid content of* C. sorokiniana* was to increase when the NaNO_3_ was depleted at 50, 100, and 200 *μ*mol·m^−2^·s^−1^; however, the final lipid content for 50, 100, and 200 *μ*mol·m^−2^·s^−1^ was only 15.28%, 14.84%, and 10.42%, respectively (Figure 3(c)), which was lower than initial lipid content. These predictions may imply that, when the growing environment is comfortable for microalgae, the photosynthate is mainly used for growth. Both biomass and lipid content for* C. sorokiniana* are low under phototrophic condition, which may imply that the* C. sorokiniana* is not suitable for phototrophic cultivation, or the growth medium may be a bottleneck for growth or lipid accumulation [38].

### 3.4. Neutral Lipid Productivity of HA-1 and* C. sorokiniana*

**Figure 4:**
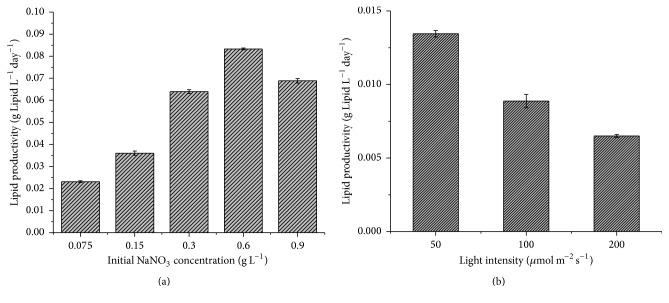
The lipid productivity of* Coelastrum* sp. HA-1 (a) under various initial NaNO_3_ concentrations from 0.075 to 0.9 g·L^−1^ after 24 days and of* C. sorokiniana* (b) under various light intensities supplemented from 50 to 200 *μ*mol·m^−2^·s^−1^ after 7 days.

As demonstrated in Figure 4, the maximum neutral lipid productivity of HA-1 was 0.083 g·L^−1^·day^−1^ for 0.6 g·L^−1^ initial nitrogen concentration at 24th day. For 0.075, 0.15, 0.3, and 0.9 g·L^−1^ the neutral lipid productivity was 0.023, 0.036, 0.064, and 0.069 g·L^−1^·day^−1^, respectively. The lipid productivity of* C. sorokiniana* was only 0.013, 0.0089, and 0.0065 g·L^−1^·day^−1^ at 50, 100, and 200 *μ*mol·m^−2^·s^−1^, respectively.

### 3.5. Sensitivity Analysis

**Figure 5:**
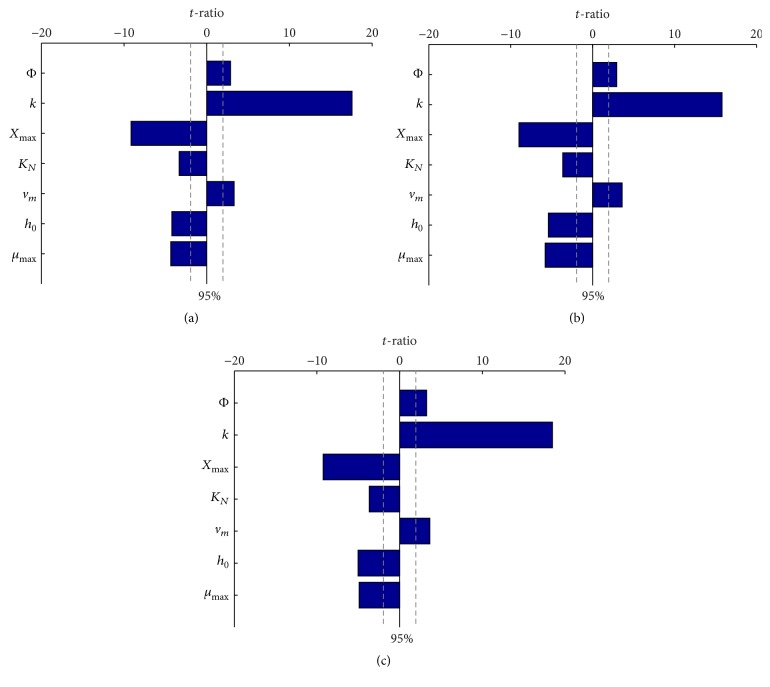
Sensitivity of model inputs without parameter *c* (carbohydrate content) for* C. sorokiniana* under 50 (a), 100 (b), and 200 (c) *μ*mol·m^−2^·s^−1^. Model inputs were altered by ±20% with lipid content after 24 days compared with baseline lipid content output. Vertical dash lines represent 95% confidence interval.

As showed in Figure 5, a sensitivity analysis was performed by increasing and decreasing each input by 20%, then estimating *t*-ratios of each input parameter for the variance of lipid content of* C. sorokiniana* using analysis of variance to the model. Sensitivity results for 50, 100, and 200 *μ*mol·m^−2^·s^−1^ were presented in Figures 5(a), 5(b), and 5(c), respectively.

The *t*-ratio value of parameter *c* (carbohydrate content) in (6) was not shown in Figure 5, because the relationship between parameter *c* and lipid content was linear. It was sensitive for the model when the parameter value of carbohydrate content changed. The *t*-ratio values of parameter *c* were 1.131 × 10^16^, 5.796 × 10^15^, and +*∞* for 50, 100, and 200 *μ*mol·m^−2^·s^−1^, respectively. Hence, the value of parameter *c* should be firstly determined by mass balance or experiment when the model is applied in other algal species.

For all of light conditions, variables associated with nitrogen-to-protein conversion factor, Baranyi-Roberts parameter, and the maximum algal concentration have a significant effect on lipid accumulation. The model is insensitivity to variance of lipid content in some parameters such as the parameter Φ under different light conditions.

Those variables with a *t*-ratio greater than the *t*-ratio at 95% confidence interval have a large effect on the model outputs and thus need to be obtained more accurately than those in this interval when adapting the model to other algal species.

## 4. Conclusion

Lipid content has been modeled by a linear function of nitrogen quota of cell, and the growth of microalgae has been simulated by a kinetic model at different initial nitrogen concentrations and light intensities. The model was validated in HA-1 and applied in* C. sorokiniana*. After sensitivity analysis for the model, carbohydrate content, nitrogen conversion factor, and the maximum algal concentration were significant effect on lipid accumulation. Upregulating initial nitrogen concentrations helped in producing higher biomass, and low nitrogen levels accelerated lipid accumulation. Thus, multiscale regulation of nitrogen was beneficial to get higher lipid productivity in commercial scale.

## Figures and Tables

**Table 1:** Simulation parameter values (a) and simulation *R*^2^ (b) under different initial concentrations of NaNO_3_ for HA-1.

Parameter	Initial NaNO_3_ concentration [g L^−1^]				
	0.075	0.15	0.3	0.6	0.9
(a) Parameter values					
*μ*_max_	0.313 ± 0.049	0.340 ± 0.050	0.295 ± 0.064	0.300 ± 0.012	0.264 ± 0.012
*h*_0_	−0.624 ± 0.329	−0.719 ± 0.292	−1.338 ± 0.436	−1.289 ± 0.087	−1.593 ± 0.156
*v*_*m*_	0.298 ± 0.077	0.156 ± 0.001	0.623 ± 0.181	0.410 ± 0.019	0.285 ± 0.045
*K*_*N*_	0.002 ± 0.000	0.003 ± 0.001	0.142 ± 0.025	0.327 ± 0.013	0.198 ± 0.035
*X*_max_	1.454 ± 0.006	2.288 ± 0.122	4.094 ± 0.226	5.946 ± 0.245	6.738 ± 0.098
*k*	2.119 ± 0.000	2.121 ± 0.003	2.123 ± 0.001	2.122 ± 0.004	2.119 ± 0.007
*c*	0.436 ± 0.059	0.440 ± 0.025	0.440 ± 0.023	0.440 ± 0.009	0.440 ± 0.002
Φ	0.052 ± 0.040	0.050 ± 0.020	0.050 ± 0.037	0.049 ± 0.006	0.050 ± 0.024

(b) Simulation *R*^2^					
*X*(*t*)	0.9999	0.9925	0.9667	0.9794	0.9858
*N*(*t*)	1.0000	1.0000	0.9973	0.9999	1.0000
*L*(*t*)	0.9995	0.9999	0.9994	0.9989	0.9994

*α*(*t*)	$\frac{1.8664}{\exp\left(0.1953t/\left(0.313t-0.624\right)\right)}$	$\frac{2.0524}{\exp\left(0.2445t/\left(0.340t-0.719\right)\right)}$	$\frac{3.8114}{\exp\left(0.3947t/\left(0.2950t-1.338\right)\right)}$	$\frac{3.6292}{\exp\left(0.3867t/\left(0.300t-1.289\right)\right)}$	$\frac{1.8664}{\exp\left(0.4206t/\left(0.264t-1.593\right)\right)}$

**Table 2:** Simulation parameter values at different light intensities for *C. sorokiniana*.

Parameter	Light intensity [*μ*mol·m^−2^·s^−1^]		
	50	100	200
*μ*_max_	0.466	0.297	0.455
*h*_0_	−1.493	−1.910	−1.559
*v*_*m*_	0.285	0.285	0.285
*K*_*N*_	0.198	0.198	0.198
*X*_max_	1.009	1.228	0.678
*k*	0.735	0.775	0.746
*c*	0.113	0.093	0.102
Φ	5.794*E* − 07	3.450*E* − 03	2.670*E* − 04
